# Is Autophagy Involved in Pepper Fruit Ripening?

**DOI:** 10.3390/cells9010106

**Published:** 2020-01-01

**Authors:** Omar López-Vidal, Adela Olmedilla, Luisa María Sandalio, Francisca Sevilla, Ana Jiménez

**Affiliations:** 1 Department of Stress Biology and Plant Pathology, CEBAS-CSIC, Murcia 30100, Spain; omalopez17@yahoo.com (O.L.-V.); fsevilla@cebas.csic.es (F.S.); 2 Department of Biochemistry, Cellular and Molecular Biology of Plants, EEZ-CSIC, Granada 18160, Spain; adela.olmedilla@eez.csic.es (A.O.); luisamaria.sandalio@eez.csic.es (L.M.S.)

**Keywords:** autophagy, *Capsicum annuum*, fruit ripening, pepper, organelles, oxidative stress, vacuolar vesicles

## Abstract

Autophagy is a universal self-degradation process involved in the removal and recycling of cellular constituents and organelles; however, little is known about its possible role in fruit ripening, in which the oxidation of lipids and proteins and changes in the metabolism of different cellular organelles occur. In this work, we analyzed several markers of autophagy in two critical maturation stages of pepper (*Capsicum annuum* L.) fruits where variations due to ripening become clearly visible. Using two commercial varieties that ripen to yellow and red fruits respectively, we studied changes in the gene expression and protein content of several autophagy (ATG) components, ATG4 activity, as well as the autophagy receptor NBR1 and the proteases LON1 and LON2. Additionally, the presence of intravacuolar vesicles was analyzed by electron microscopy. Altogether, our data reveal that autophagy plays a role in the metabolic changes which occur during ripening in the two studied varieties, suggesting that this process may be critical to acquiring final optimal quality of pepper fruits.

## 1. Introduction

Fruit ripening is a very dynamic developmental process which involves dramatic changes in the fruit’s properties. In peppers, this process includes fruit changes in color, sugar accumulation, or flesh softening [1,2]. The recycling of molecules and organelles is likely required in this complex process in order to reduce the energy requirements for the plant. Autophagy is a highly-conserved, intracellular, self-degradation system in eukaryotes for the removal and recycling of cellular components. In plants, two types of autophagy, micro- and macro- autophagy, have been described [3]. Both processes can be either nonselective or selective in the removal of specific organelles such as chloroplasts, mitochondria, or peroxisomes. 

During microautophagy, it is known that some organelles or portions of cytoplasm are invaginated by a vacuolar membrane, forming intravacuolar vesicles which are then digested by vacuolar hydrolases [4]; however, the mechanisms involved in microautophagy regulation in plants have not been established, and as far as we know, there are no specific markers of this process. On the other hand, during macroautophagy (generally termed as “autophagy”), cells create double membranes called autophagosomes which engulf organelles or portions of the cytoplasm and fuse with the tonoplast to release the internal-vesicle material inside the vacuole for breakdown by resident hydrolases. Autophagosome formation relies on extensive membrane rearrangements, and is mediated by the concerted action of a set of autophagy-related proteins (ATG) [5]. 

More than 30 *ATG* genes have been reported in Arabidopsis and other plants including tobacco, rice, maize, and pepper [6,7,8], but information about the possible involvement of autophagy in the development of fruit is very scarce. Analyses of gene expression have shown that autophagy plays an important role in nutrient recycling and plant stress tolerance [9,10]. The detection of most of the *ATG* genes initially identified in yeast and then in practically all studied eukaryotes points to a highly-conserved nature of the autophagy process. Autophagosome formation involves ATG8–phosphatidylethenolamine (ATG8–PE) conjugates targeted to the autophagosomal membranes, with this lipidated form being related to the number of autophagosomes [11]. Among the *ATG* genes, *ATG8* codifies for the central protein involved in autophagy, and nine isoforms have been identified in Arabidopsis (*ATG8a* to *ATG8i*), while only five (from a to e) have been described in pepper plants [7]. The ATG9 protein complex involves ATG9, ATG2, and ATG18, and it participates in the recruitment of lipids, while ATG3–ATG4ab–ATG7–ATG8a-i and ATG12ab–ATG5–ATG7–ATG10 participate in the phagophore expansion and enclosure. ATG4 is the sole protease among ATG proteins with a dual role in the regulation of autophagy through its relationship with ATG8. ATG4 first processes ATG8 for subsequent lipidation, but it is also responsible for the delipidation or cleavage of ATG8-PE [12]. Other components of the autophagy process are cargo receptors, such as the NBR1 (NEIGHBOR OF BRCA1) protein, that in Arabidopsis binds to autophagosomal-bound ATG8, targeting ubiquitinated proteins for autophagic degradation [11]. 

As mentioned, autophagy can degrade mitochondria, peroxisomes, chloroplasts, proteasome, ribosomes, endoplasmic reticulum, and other cell organelles in plant cells under specific conditions [5]. Although not fully understood, selective autophagy was initially described as a housekeeping process, although recent studies have demonstrated its involvement in stress tolerance and development. Moreover, pexophagy has been shown in highly-oxidized peroxisomes, in those lacking an active LON2 (LON protease homolog 2, peroxisomal) protease [13], and in response to heavy metal stress [14]. In eukaryotes, LON isoforms are also found in mitochondria (LON1) and chloroplasts (LON4), and the absence of LON1 in KO mutants provokes an aberrant mitochondrial morphology and a modification of the respiratory function and plant performance [15]. Although all these autophagy key molecules have been extensively studied in model plants, their role in crop plants remains elusive [16]. 

Pepper (*Capsicum annuum* L.) is one of the most widely-consumed vegetables due to its nutritional value (i.e., it is rich in ascorbic acid (vitamin C), β-carotene (provitamin A), flavonoids, carotenoids, etc.) and the diversity of its culinary roles (condiments, spices, conserves, etc.) [1,2]. Bell/sweet peppers are mainly classified into three types: California, Lamuyo, and Dulce italiano. California fruits, used in this study, change color to either red or yellow after ripening, depending on the variety, with green peppers being the least ripe edible fruits. Fruit ripening is a genetically-programmed and highly-coordinated process which involves changes in color (loss of green and increase in nonphotosynthetic pigments), taste (increase in sugar and decrease in organic acids), firmness (softening), and flavor (the production of volatile compounds). All these new characteristics make the fruit attractive for other organisms to promote seed dispersion [17,18]. 

In addition to their unique colors, each bell pepper has clear nutritional benefits: green immature peppers have plenty of chlorophyll, and mature fruits different carotenoids; yellow fruits contain more lutein and zeaxanthin; orange peppers have more alpha-, beta-, and gamma-carotene; and mature red ones more lycopene and astaxanthin [19]. During fruit ripening, the production of reactive oxygen species (ROS) plays an important role, as in the conversion of chloroplasts to chromoplasts, or the deterioration in the quality or the appearance of flavors and disagreeable scents, or the destruction of vitamins [1,20]. It has been reported that pepper fruit ripening and the harvest period influence the antioxidant content and the development of oxidative processes in different varieties, with increases in oxidative stress markers like lipid peroxidation and carbonyl oxidation, which could involve the degradation and recycling of peroxisomes in the cells where these processes take place [21]. 

On account of this agro-economic value, growing attention is being paid to gaining a better understanding of the molecular changes associated with pepper fruit ripening. In this sense, it would be useful to have more information on the roles that micro- and/or macro- autophagy may play in this developmental process, considering the synthesis and recycling metabolisms taking place in the fruits. These reasons, and the very scarce information about autophagy in nonmodel plants, prompted us to determine the occurrence of autophagy in pepper fruits in two commercial and optimal consuming ripening stages, analyzing different markers in two pepper varieties, i.e., Galena and Alonso, maturing to different colors, i.e., yellow and red, respectively.

## 2. Materials and Methods

### 2.1. Plant Material and Growth Conditions

Sweet California pepper fruits (*Capsicum annuum* L. vars. Galena and Alonso) were grown by Syngenta seeds S.A. (El Ejido, Almería, Spain) [22]. Fruits were harvested at two optimal commercial stages: immature green and mature yellow (for Galena variety) and red (for Alonso variety). 

### 2.2. Crude Extracts 

Extraction was carried out at 0–4 °C. For enzymatic analyses, pepper fruit pericarps were ground in a mortar and pestle in the presence of an extraction buffer (50 mM Tris-HCl pH 7.5, 0.1 mM EDTA, 0.1% (*v*/*v*) Triton X-100, 10% (*v*/*v*) glycerol, 0.5 mM PMSF (phenylmethanesulfonyl fluoride) and 2 mM DTT (dithiothreitol) Sigma (Merck, Darmstadt, Germany) in a 1:1 (*w*/*v*) ratio, and further filtered through four layers of nylon cloth. The homogenates were then centrifuged at 16,000× *g* for 25 min and 4 °C, and the supernatants were used for the activity assays. 

For Western blotting assays, pepper fruit pericarps were homogenized in liquid N_2_ and extracted in a medium containing 50 mM Tris-HCl, pH 7.5, 0.1 mM EDTA, 0.1% (*v*/*v*) Triton X-100, 5 mM DTT, 0.1 mM PMSF (except for LON proteins detection) and 10% (*v*/*v*) glycerol. Homogenates were filtered through four layers of nylon and centrifuged at 16,000× *g* for 15 min. Supernatants were used for protein precipitation with three volumes of 100% acetone at −20 °C overnight. The mixture was then centrifuged at 10,000× *g* for 10 min at 4 °C. The precipitate, which contained most of the proteins, was resuspended in 0.5 mL of 50 mM Tris-HCl, pH 7.5. When necessary, the supernatants were concentrated using Amicon 3K filters (Millipore-Merck, Darmstadt, Germany), following the manufacturer’s instructions. 

### 2.3. SDS–PAGE and Western Blot

SDS–PAGE was carried out on 15% polyacrylamide gels [16]. For Western blot analyses, 40 µg of protein from fruit extracts were transferred to nitrocellulose membranes using a semidry Trans-Blot cell (Bio-Rad, Hercules, CA, USA). The membranes were used for cross-recognition assays using polyclonal antibodies anti-CrATG4 (Agrisera, Vännäs, Sweden), anti-AtATG5 (Agrisera), anti-AtATG8a (Abcam, Cambridge, UK), anti-AtNBR1 (Agrisera), anti-HsLON1 (Sigma, Merck)), and anti-AtLON2, generously donated by Dr. Akira Kato from Niigata University (Niigata, Japan) (1:8000 dilution) or monoclonal 6His antibody (Agrisera). For immunodetection in membranes, a goat anti-rabbit or goat anti-mouse IgG–horseradish peroxidase conjugate (Santa Cruz Inc., Dallas, TX, USA) was used as the secondary antibody, together with an enhanced chemiluminescence kit (Thermo Fisher, Waltham, MA, USA). Densitometry of the different bands was performed using an image analyzer (Amersham Imager 600, GE, Boston, MA, USA) and the ImageQuanTL 8.1, Program (Amersham, USA). Densitometry of the Pounceau staining of the different bands in each lane of the membranes was used to correct the loading. 

### 2.4. Quantitative Real Time PCR (qRT-PCR)

Quantitative Real Time PCR was performed for the study of the expression of the *ATG*, *LON*, and *NBR1* genes in the fruits of pepper plants. Three biological replicates were analyzed in three different experiments. Fruit pericarp was collected, placed immediately in liquid N_2_, and stored at −80 °C. Total RNA was extracted from each tissue sample using an RNeasy Plant Mini Kit (Qiagen Iberica, Madrid, Spain) following the manufacturer’s protocol. cDNA was obtained from RNA subjected to reverse transcriptase reactions using hexamer random primers and M-MLV Reverse Transcriptase (Applied Biosystem, Waltham, MA, USA) followed by RNase H (Thermo-Fisher Scientific, USA) treatment according to the manufacturer’s instructions.

RT-qPCR was performed on a 7500 Real Time PCR System (Applied Biosystems, USA) with SYBR Green Supermix (Bio-Rad, Madrid, Spain) [23]. Each reaction was performed in triplicate. The specific primers for the RT-qPCR analyses were designed according to the target sequences from the Pepper Genome database (http://peppergenome.snu.ac.kr/blast.php); they are listed in Supplementary Table S1. The *ACTINE-100* gene (GeneBank: AY572427.1) was used as an internal control to normalize the data, since it did not undergo any change during development and ripening [22]. Relative expression (ΔCT) was calculated using the comparative CT method [24].

Relative quantity of sample (gene x) = 2[CT (control) − CT (sample)]

### 2.5. Protein Content 

Protein was measured by the protein dye-binding bicinchoninic acid method (BCA Pierce^TM^ Protein Assay Kit, Thermo-Fisher Scientific, Waltham, MA USA) using bovine serum albumin as a standard, following the manufacturer’s instructions.

### 2.6. Lipid Peroxidation and Protein Oxidation 

The extent of lipid peroxidation in pepper fruit pericarps was estimated by determining the concentration of thiobarbituric acid reactive substances (TBARS), and the dinitrophenyl hydrazine (DNPH) method was followed for the determination of protein oxidation (carbonyl protein content) [21]. 

### 2.7. Malate Synthase Activity

The fruit homogenate was obtained from fruit pericarps using a mortar and pestle after passing twice through four nylon layers. The material was then centrifuged at 27,000× *g*. An enzyme assay was conducted at room temperature in a Jasco V-630 spectrophotometer (Jasco, Easton, MD, USA). Malate synthase (MS) (l-malate glyoxylate-lyase CoA-acetylating EC (4.1.3.2)) was assayed following the increase in absorbance at 412 nm due to the glyoxylate-dependent production of 5-thio-2 nitrobenzoic acid [25]. 

### 2.8. Cytochrome C Oxidase Activity

Cytochrome c oxidase (CCO) activity was measured following the decrease of absorbance at 550 nm due the oxidation of the cytochrome C [18]. 

### 2.9. In Vivo ATG4 Cleavage Assay

Measurement of ATG4 activity was carried out by the electrophoretic separation of cleaved ATG8 substrate containing a C-terminal tag after the cleavage site. For this, a recombinant human GABARAP protein (Fc Chimera, ABCAM: ATG8-Fc) also containing an N-terminal 6His tag was used. Then, 100 µg of protein extract was incubated with recombinant 6His ATG8-Fc (0.6 µg) in TBSK reaction buffer (50 mM Tris, 140 mM NaCl, 30 mM KCl pH 8) and 100 µM DTT at 30 °C for 45 min. The reaction was stopped by the addition of sample buffer (β-mercaptoethanol-free Laemmli buffer) and 7 min of boiling, after which the samples were resolved on 15% SDS–PAGE and subsequently analyzed by Western blot, using anti-6His monoclonal antibody. A human HsATG4B recombinant protein (Abcam, Cambridge, UK) was used as positive control, and an extract of Galena immature fruit without the Fc chimeric substrate as negative one. 

### 2.10. Electron Microscopy 

Pepper fruit pieces (2 mm^2^) taken from the pericarp (Figure S1) were fixed in 0.2% (*v*/*v*) glutaraldehyde and 4% (*w/v*) paraformaldehyde in 50 mM PIPES-KOH buffer (pH 7.4) for 1 h at 4 °C and were dehydrated through a graded ethanol series (30–100%, *v*/*v*) and infiltrated with LR White resin [26]. Sections were poststained in uranyl acetate and examined in a Zeiss EM 10C electron microscope (Carl Zeiss AG, Oberkochen, Germany). 

### 2.11. Sampling and Statistical Analysis

Three fruits per variety were chosen for each extract, and at least two different extracts were obtained in three independent experiments. The significance of any differences between mean values was determined by one-way analysis of variance; the comparative analysis used was the Kruskal-Wallis’ test (*p* < 0.05).

## 3. Results

### 3.1. Oxidative Damage Determinations

**Figure 1:**
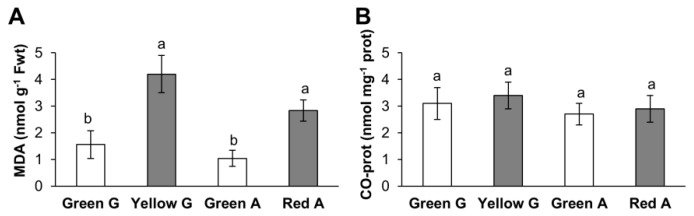
(**A**) Lipid peroxidation (malondialdehyde MDA content) and (**B**) carbonyl (CO) protein content in immature green Galena (G), mature yellow Galena, green Alonso (A) and mature red Alonso pepper fruits. Data are means ± standard error (SE) of three technical replicates of at least three biological samples. Different lower case letters indicate that data are significantly different according to the Kruskal-Wallis test at *p* < 0.05.

To investigate the possible oxidative damage produced after the pepper ripening, lipid peroxidation and protein carbonylation were compared between the green immature and the yellow or red mature peppers of the corresponding varieties. MDA, as indicative of oxidative damage to lipids, presented higher values in the mature fruits of the two varieties compared to immature ones (Figure 1A). However, no significant changes in the content of carbonylated (CO) proteins were observed among the fruits (Figure 1B). 

### 3.2. Expression Levels of Autophagy Markers 

**Figure 2:**
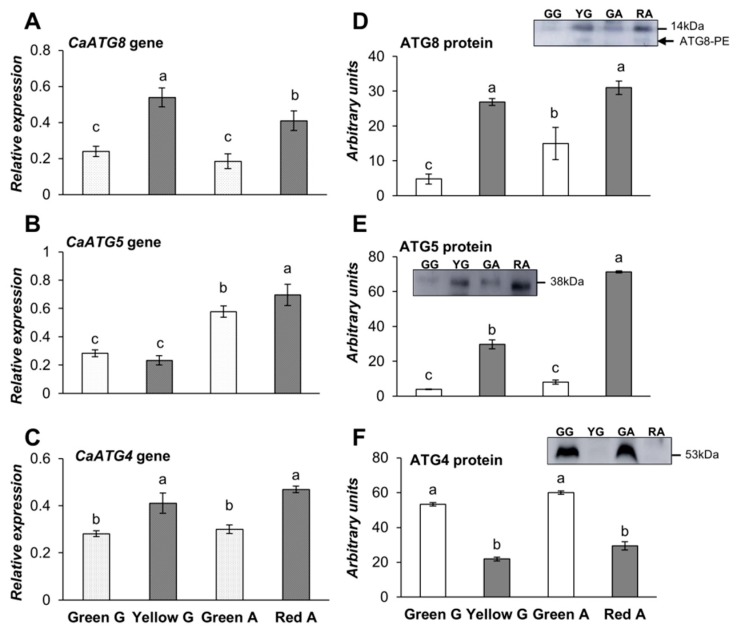
Expression analysis of (**A**) *CaATG8a* gene (**B**) *CaATG5* gene and (**C**) *CaATG4* gene relative to *ACTINE-100* by RT-qPCR; Western blot analysis of the (**D**) CaATG8 (14 kDa protein; arrow points the lipidated phosphatidylethanolamine: PE form), (**E**) CaATG5 and (**F**) CaATG4 proteins in green Galena (GG), yellow Galena (YG), green Alonso (GA), and red Alonso (RA) pepper fruits. Data are means ± standard error (SE) of three technical replicates of three biological samples. Different lower case letters indicate that data are significantly different according to the Kruskal-Wallis test at *p* < 0.05.

To determine whether autophagy is involved in pepper fruit ripening, we studied whether ATG gene expressions are changed in the stages studied. For that, we quantified the transcripts levels of *CaATG8a, CaATG4, CaATG5*, and *CaATG9* by real-time quantitative PCR (q-PCR). Autophagy marker ATG8 protein, which binds to autophagosomal membranes and remains associated with the completed vesicles until their lytic destruction in the vacuole, was also monitored in the green pepper fruits of both varieties, as well as in the yellow Galena and red Alonso ripened varieties. *CaATG8a* gene expression showed a significant increase in both yellow and red peppers, indicating that autophagy is associated to ripening (Figure 2A). ATG5, involved in phagophore expansion, showed a significant increase of transcript *CaATG5* only in red pepper fruits (Figure 2B). *CaATG4* gene expression was higher in both mature fruits (Figure 2C). The analysis of the different ATG proteins was carried out by Western blot, and a quantification of the immunodetected respective band was carried out. ATG8 protein (Figure 2D) displayed a marked enhancement in both yellow and red pepper fruits, in accordance with transcriptomic results. The lipidated form (phosphatidylethanolamine: PE adduct) was detected mainly in yellow and red ripened fruits (arrow in Figure 2D). We also identified ATG5 protein, showing a higher content in the yellow and red fruits (Figure 2E). However, ATG4, the cysteine protease involved in the lipidation/delipidation of ATG8, decreased in these ripened fruits, in spite of its mentioned transcription increase (Figure 2F). 

**Figure 3:**
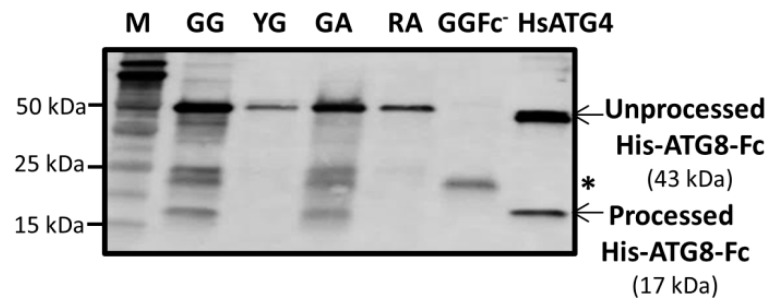
ATG4 cleavage activity assay in extracts of green Galena (GG), yellow Galena (YG), green Alonso (GA), and red Alonso (RA) pepper fruits. Recombinant 6His-ATG8-Fc substrate and 100 µM DTT were added to fruit extracts and incubated for 20 min before being subjected to SDS-PAGE and Western blot using anti-6His monoclonal antibody. The unprocessed 43 kDa substrate and processed ~17 kDa product are indicated. A negative control is included using GG fruits without 6His-ATG8-Fc (GGFc-) in the assay, as well as a positive control using human recombinant HsATG4B protein instead of fruit extract. The unspecific band at 30 kDa is also pointed (asterisk).

This prompted us to analyze the ATG4 activity in green, yellow, and red fruits. Full-length noncleaved and cleaved byproducts can be monitored by separating them on SDS/PAGE followed by immunoblotting. The His-ATG8-Fc synthetic substrate was used to investigate ATG4 cleavage activity in the pepper protein samples. Measurement of ATG4 activity in the pepper extracts using an anti-His antibody revealed a higher cleavage activity in the green fruits form Galena (GG) and Alonso (GA) than in the ripened ones (YG and RA), as evidenced by the presence of an around 17 kDa protein band (processed pATG8-Fc) in the Western blot (Figure 3). Several bands appeared in the unripened fruits in which both the noncleaved product and the cleaved one were highly detected, indicating a higher activity than in the mature fruits. Less ATG8-Fc substrate remained unprocessed in the mature fruits, in spite of the lower 17 KDa product. A negative control of green Galena fruit extract without the ATG8-Fc substrate (GGFc-) was performed, revealing that the signal around 30 kDa appearing in green fruits was a nonspecific band or an endogenous protein recognized by the anti-His antibody, indicating the absence of the processed product. A positive control was also included using human HsATG4B protein instead of fruit extract.

**Figure 4:**
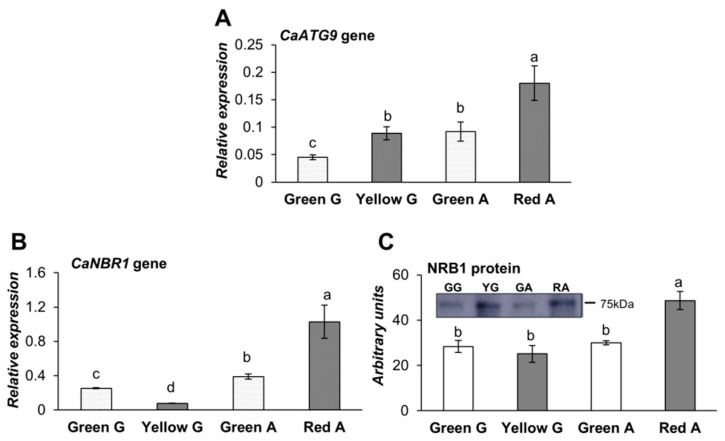
Expression analysis of (**A**) *CaATG9*, (**B**) *CaNBR1* genes relative to *ACTINE-100* by RT-qPCR, and (**C**) Western blot analysis of the CaNBR1 protein in green Galena (GG) and yellow Galena (YG), green Alonso (GA), and red Alonso (RA) pepper fruits. Data are means ± standard error (SE) of three technical replicates of three biological samples. Different lower case letters indicate that data are significantly different according to the Kruskal-Wallis test at *p* < 0.05.

The level of gene expression of *CaATG9* was also studied due to its involvement in autophagosome formation; it was found that it followed a similar pattern to *ATG8* and *ATG4*, increasing in the mature fruits (Figure 4A). The protein expression was not evaluated due to the lack of antibodies against this protein. 

**Figure 5:**
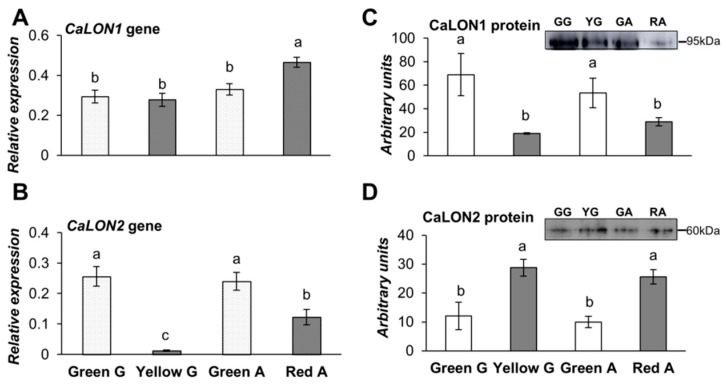
Expression analysis of (**A**) *CaLON1* and (**B**) *CaLON2* genes relative to *ACTINE-100* by RT-qPCR, and Western blot analysis of the (**C**) CaLON1 and (**D**) CaLON2 proteins in green Galena (GG), yellow Galena (YG), green Alonso (GA), and red Alonso (RA) pepper fruits. Data are means ± standard error (SE) of three technical replicates of three biological samples. Different lower case letters indicate that data are significantly different according to the Kruskal-Wallis test at *p* < 0.05.

The analysis of NBR1, a cargo adaptor between the damaged material and the autophagosome, showed a significant increase in its gene and protein expression in the red pepper (Figure 4B,C, respectively), while in yellow fruits, the gene expression decreased while the protein level was maintained. The analysis of the gene expression of the LON proteases revealed a higher level of mitochondrial *CaLON1* in red fruits (Figure 5A), while the peroxisomal *CaLON2*, decreased significantly in both ripened yellow and red fruits related to the green peppers (Figure 5B). Notably, their protein contents followed opposite patterns to that of the respective gene expression (Figure 5C,D).

### 3.3. Malate Synthase and Cytochrome C Oxidase Activities

**Figure 6:**
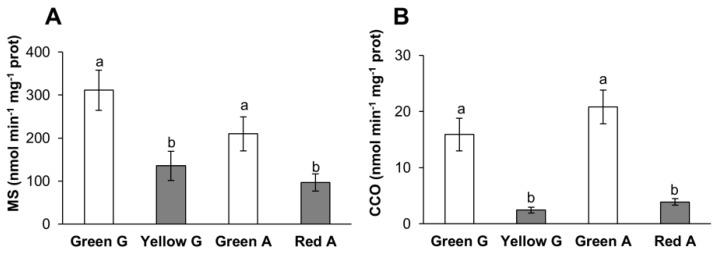
Enzymatic specific activity of (**A**) malate synthase (MS) and (**B**) cytochrome c oxidase (CCO) in green Galena (G), yellow Galena, green Alonso (A), and red Alonso pepper fruits. Data are means ± standard error (SE) of three technical replicates of three biological samples. Different lower case letters indicate that data are significantly different according to the Kruskal-Wallis test at *p* < 0.05.

An analysis of the activity of malate synthase (MS) and cytochrome c oxidase (CCO) enzymes, which have been reported to be degraded by peroxisomal and mitochondrial LON protease families, respectively, indicated that both enzymes showed higher activities in green pepper fruits than in ripened yellow and red ones (Figure 6A,B).

### 3.4. Detection of Vesicles Containing Organelles

**Figure 7:**
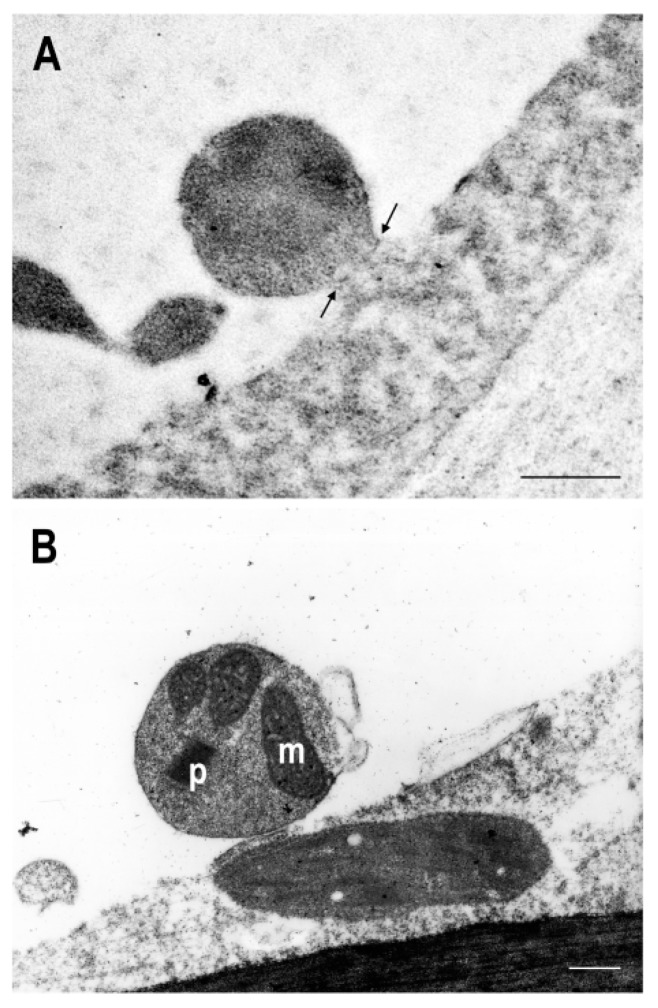
Green pepper var. Galena. Details of vesicles in the vacuole: (**A**) formation of the vesicle still connected to the cytoplasm (arrows) and (**B**) vesicle containing different organelles, mitochondria (m) and peroxisome (p). Scale bar = 500 nm.

**Figure 8:**
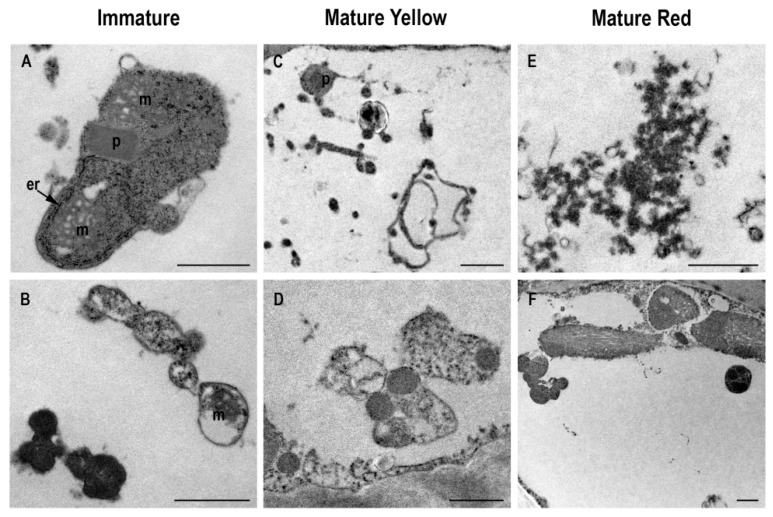
Electron micrographs of green Galena (**A**,**B**), yellow Galena (**C**,**D**) and red Alonso (**E**,**F**) pepper fruits. p = peroxisome, m = mitochondria, er = endoplasmatic reticulum. Scale bar = 1 µm.

To gain more insights into the role of autophagy in the ripening of pepper fruits, we studied the formation of possible autophagic vesicles by analyzing the ultrastructure of the pericarp cells of pepper fruits (Figure S1) by electron microscopy. Different types of vesicles were observed inside the vacuoles in different pepper fruits; some of these vesicles contained cytosolic material, while others contained several organelles such as peroxisomes or mitochondria (Figure 7 and Figure 8). These vesicles measured between 1 and 3 microns, and shown in Figure 7, they could be transported to the vacuole by the invagination of the tonoplast. Another type of vesicles was dense in electrons, and looked like aggregations of several vesicles (Figure 8B). Yellow peppers showed less content of vesicles than green peppers (Figure 8A–D), and in red peppers, the vacuole was even emptier than in yellow ones (Figure S1 and Figure 8E,F) showing some amorphous material which probably occurred as the result of degradation (Figure 8E). To try to identify autophagosomes, immunocytochemistry using antiATG8a antibodies, a marker of macroautophagy, was carried out; however, no specific labeling was observed in the vesicles containing different organelles. As suggested by the tonoplast engulfment, some of these vesicles could be related to microautophagy; however, this is difficult to demonstrate due to the lack of specific markers for this process.

## 4. Discussion

Autophagy regulates cell development, senescence, and response to different biotic and abiotic stresses, although the nature of plant cargos and the mechanisms involved in the specificity of this process remain unclear. Little is known about the functioning of autophagy during fruit development and ripening, and studies of the process through analyses of *ATG* genes or ATG proteins in pepper fruits are quite scarce [6]. It has been reported that the transcriptional upregulation of *ATG* genes is a marker of enhanced autophagy [27]. We observed an increase in gene expression of *ATG8a* in ripened pepper fruits, and also found that *ATG4* and *ATG9* gene expression increased in both red and yellow ripened fruits, which is indicative of autophagic activity in these fruits. However, *ATG5*, *NBR1*, and *LON1* increased only in the red fruits of the Alonso var., suggesting that a different mechanism in the two studied varieties could be activated during ripening. The parallel behavior between the protein content measured by Western blot and gene expression found in the cases of ATG8, ATG5, and NBR1, but not for ATG4 (which presented an opposite pattern), possibly points to the posttranscriptional regulation of this latter protein or a degradative process on ATG4, but it reinforces the idea that autophagy participates in ripening. It is interesting to note that unlike in Arabidopsis, rice, maize, and wheat, which present two *ATG4* genes, only one was identified in pepper, as previously described in tobacco [9], suggesting a “solanaceae crop-specificity” in the number of *ATG4* genes [7]. The participation of autophagy has been shown in pepper leaves in response to drought, salinity, heat, and cold stresses, with changes in the expression of different *ATG* genes including *ATG4* [7]. Among ATG proteins, ATG4 cystein protease cleaves ubiquitin-like ATG8 proteins, which provokes the exposure of conserved glycine residue at the C terminus. The processed ATG8 conjugates to the phagophore membrane through lipidation with PE, which is then delipidated by ATG4 activity [28]. Lipidated ATG8 protein has been described to accumulate during human cell starvation [29], and the delipidation activity of ATG4, more than the initial cleavage of ATG8-like proteins, has been shown to be the main target of the regulation of the autophagy process [30]. In this work, the cleavage activity was used to assay ATG4 processing activity on a quimeric ATG8 substrate. The lower activity of ATG4 observed in ripened peppers could then regulate the accumulation of ATG8, and in fact, lipidated ATG8-PE content was found to be higher in ripened pepper fruits. The physiological significance of the lower delipidation activity of ATG4 is not conclusively elucidated. The inactivation of ATG4 by ROS at the site of autophagosome generation has been shown to promote ATG8 lipidation in this way, allowing autophagy progression [30]. On the other hand, it has been shown that the deconjugation of ATG8 occurs in two types of membranous structures, i.e., PAS and nonPAS, with different roles in autophagy [31], and that during this process, the delipidation reaction of ATG8-PE by ATG4 plays an important role in the efficient expansion of isolation membranes other than supplying nonlipidated ATG8 [32]. It is possible then that the lower amount of ATG4 protein, together with the reduced processing activity detected in ripened pepper fruits and more ATG8-PE protein detected, may be related to the less autophagic structures found in these fruits compared to immature ones, in which autophagy through the ATG4 pathway is activated, as evidenced by a higher level of protein and processing activity. Moreover, the distinct protein pattern observed in the cleavage assay in the unripened peppers could represent a different processing activity in these fruits, although this does not preclude the existence of other proteases cutting the chimeric ATG8 in a nonspecific way; this question merits deeper investigation. On the other hand, it seems that different proteases may also be acting in the extract from the mature yellow and red fruits, since the intensity of the 43 kDa in that lane is much lower than in the green fruits and in the positive control lane (HsATG4). In fact, a very high level of protease activity has been described in red peppers, which was not inhibited by PMSF [33]. It has been established that during nutrient starvation in animal cells, superoxide production regulates autophagy, and in yeast, ROS directly regulates the ATG4 protein, thus activating autophagy [34]. More recently, ATG4 has been shown to be redox regulated in *Chlamydomonas* [35]. The role of ROS as signal molecules during development and biotic/abiotic stresses in plants, as well as other organisms, is well known [36,37,38]. During fruit ripening, an increase in ROS production and their oxidized products as lipids or proteins has been evidenced in pepper fruits in different varieties and under changing environmental growth conditions [1,21], which could regulate the autophagy process through its impact on oxidative-regulated proteins such as ATG4. In fact, in our experiments, the increased lipid peroxidation in ripened fruits is indicative of oxidative stress, although deeper study is required to establish this possible correlation. Moreover, ripening involves huge changes in metabolism, including the chloroplast to chromoplast transition, but also changes in the proteome of mitochondria and peroxisomes [2,18,39]. In fact, protein carbonylation has been reported to increase in mitochondria from immature pepper fruits [17]. In this transition, autophagy could play an important role, not only as a defense mechanism to eliminate damaged components including oxidized proteins, but also for the recycling necessary to maintain the new metabolic processes occurring during ripening. Also, in senescence, a process accompanied by increased mitochondrial ROS generation [40], the organelles are degraded by mitophagy in Arabidopsis [41]. These data reinforce the idea that the ROS/scavengers balance is a key point in the regulation of autophagy which, in turn, may influence the functional metabolism in the cell during developmental processes and stress response in plants. Regarding ATG9, it has been shown that it is the only integral membrane ATG protein that is essential for endoplasmic reticulum (ER)-derived autophagosome formation in plants [42]. In ripened pepper fruits, *ATG9* expression increases; similar results were observed during grape berry skin ripening [43]. Selective autophagy has been shown to be essential for the quality control of peroxisomes in leaves and for adequate plant development under natural growth conditions. The number of peroxisomes was found to be higher in the leaves of *Atg5* Arabidopsis mutants, and these plants accumulated an inactive form of catalase [44]. 

The role of autophagy in pepper ripening is reinforced by the observation of different vesicles inside the vacuole, with green pepper showing the highest number and variety of vesicles, while red pepper showed a lower content. In changing from immature peppers to yellow or red fruits, several changes in metabolism occur in the different cell compartments, including the transition from chloroplasts to chromoplasts [1,2]; meanwhile, once mature fruits have reached this stage, a different autophagy mechanism may occur to maintain the fruit development. Our observation of peroxisomes inside vesicles in the vacuole in pepper fruits could be indicative of the existence of damaged or obsolete organelles, as has been reported in Arabidopsis plants [44]. Our results suggest that these organelles, together with mitochondria, could be degraded by autophagy, although we were unable to distinguish between macro- and micro- autophagy. Furthermore, taking into account that no specific microautophagy markers have been described, more research is required to clarify this issue. Recently, a chlorophagy process was reported by microscopic observations showing swollen chloroplasts which were not engulfed by autophagosome but which were partially labeled with ATG8a, and were directly engulfed by the tonoplast, as described for microautophagy [45]. In this way, this process seems to avoid the cytosolic accumulation of dysfunctional chloroplasts, a key event under stress situations as exposure to high-intensity light, ultraviolet-B, or even natural sunlight [46]. Interestingly, it was shown that selective and nonselective pexophagy pathways can be initiated in parallel, as occurs after the treatment of *Hansenula polymorpha* yeast with both nitrogen limitation and excess glucose conditions [47]. 

LON proteins are described as the chaperones involved in the recovery of misfolded proteins and as proteases for severely-damaged proteins. Peroxisomal resident protease LON2 is positioned to degrade damaged proteins in this cell compartment, and in pepper fruits, this protease is also predicted to be localized in the peroxisomes [7]. It has been proposed that LON2 can facilitate the peroxisomal matrix protein degradation during peroxisome content remodeling [48], and can also regulate pexophagy in the conversion from glyoxysomes to peroxisomes, in which the degradation of proteins as isocitrate lyase and malate synthase occurs. These two key enzymes of the glyoxylate cycle have been described as being present in peroxisomes from green and red peppers, but with a lower activity in mature fruits [16,25], i.e., similar to that found in our ripened pepper fruits. Moreover, in our case, the decrease in activity is accompanied by an increase in the protein content of LON2, in spite of the decrease in the gene expression, pointing to a degradative process in the ripened fruits. This might be explained by the low fatty acid β-oxidation found in red fruit peroxisomes, which provides the acetyl-CoA necessary for malate synthase (MS) activity [48]. Fruit ripening is accompanied by the development of unique flavors that result, in part, from increased synthesis of organic acids. In banana, malate is partly responsible for the fruit flavor, together with citrate, which increases markedly during ripening [49]. The glyoxylate cycle is also involved in malate accumulation during the early development of apricots [50], and the upregulation of *MS* gene expression was shown in immature malate-accumulating grapes and in banana [51]. 

In plants, as mentioned above, the organelle membranes can be severely damaged by ROS during the life cycle, including fruit ripening. The increase in lipid peroxidation observed in our ripened fruits is similar to that previously described in red and yellow pepper fruit extracts, depending on the variety and on the environmental conditions of growth [21], in the chromoplasts from yellow pepper fruits [15] or during tomato fruit ripening [19]. Therefore, damaged membranes might be recognized by organelle-bound protein receptors and adaptors [43]. In mammalian cells, NBR1 is involved in the recognition of ubiquitinated peroxisomal membrane proteins [52], although the specific role is yet to be demonstrated. The increased expression of *NBR1*, together with the increased protein content in red peppers, points to an autophagy-related process regulated by NBR1 in this variety. *NBR1* gene expression has been shown to be upregulated during heat stress in Arabidopsis and the protein accumulated in WT plants, but not in *atg7* mutants, demonstrating the autophagy involvement in the response to stress situations [53]. Related to this, Arabidopsis *nbr1* mutants were more susceptible to different abiotic stresses like heat, drought, salt, and oxidative stresses, and accumulated ubiquitinated substrates [54] similar to those occurring in tomato *NBR1*-silenced plants [55].

Mitochondrial resident protease LON1 is located in the mitochondrial matrix in plants, including pepper fruits. It has been proposed that AtLON1 is necessary for proper mitochondrial biogenesis during seedling development [56], and *lon1* Arabidopsis KO mutants showed a decrease in the functionality of mitochondrial respiration [57]. Oxidatively-modified proteins can lose their functionality and serve as degradation targets for proteases like LON1. In fact, we previously reported that in mitochondria isolated from pepper fruits, different carbonylated proteins were identified according to the maturation stage (green and red fruits) [17]. Among these proteins, cytochrome c oxidase (CCO) was identified in ripened fruits. The observed decrease in CCO activity in our ripened fruits could be due to oxidation, as previously described in crude extracts and isolated mitochondria from pepper fruits [56]. An increase in carbonylated mitochondrial proteins has been reported during the ripening of tomato, kiwi, peach, and pepper fruits [1,18,35,58,59]. This indicates that oxidative modification could regulate the content of proteins by degradation/synthesis pathways in which autophagy may be involved. Oxidative damage to mitochondrial components such as DNA, lipids, and proteins, can cause the trapping of entire mitochondria within autophagosomes, followed by their delivery and degradation in the central vacuole [60]. In this context, the redox-regulated human autophagic protein ATG4D is associated with mitochondria in cells treated with H_2_O_2_, suggesting the involvement of this protein in mitophagy [61]. The observed vesicles containing mitochondria inside the vacuole, mainly in immature fruit, may be indicative of selective mitophagy or microautophagy, although more research is needed to clarify this point. Information about the involvement of LON1 protease in the degradation of mitochondrial proteins such as CCO in plants is quite limited, and merits more attention due to the impact of this process on plant respiration, not only during fruit ripening, but also under plant stress response. Concerning the opposite pattern for the LON1 and LON2 gene and protein expressions found in our pepper fruits, recently, it was reported that in the fungus *Thermomyces lanuginosus*, mitochondrial and peroxisomal LON proteases have different sensitivities to H_2_O_2_, and also play opposite roles in controlling growth and development [62]. 

## 5. Conclusions

Our results indicate that autophagy could be acting in both immature and ripened pepper fruits. Changes in gene expression and the protein levels of several autophagy components such as different ATGs and NBR1, together with ATG4 activity and the accumulation of proteases LON1 and LON2 were accompanied by the presence of vesicles trapping mitochondria and peroxisomes, mainly in immature green peppers. In ripened fruits, changes in autophagy markers were accompanied by oxidative damage to lipids and a reduction of mitochondrial and peroxisomal CCO and MS activities, respectively. All the results point to a role of autophagy in the recycling of organelles and the metabolic changes occurring in green, yellow, and red ripened fruits of the two studied pepper varieties. These processes could also be regulated by LON proteases in peroxisomes and mitochondria. Further studies are needed to clearly establish whether microautophagy is involved in this process, to identify the autophagy target proteins of the organelles, and to clarify the involvement of antioxidant/redox signaling pathways in each of the fruit ripening stages, as well as the role of the different specific autophagy pathways operating in each ripening stage. This work constitutes a first approach towards elucidating the in vivo role of autophagy and the mechanism by which this process is involved in the development of fruits. This line of research may be very important for the agro-economic market and for consumers, due to the possible contribution of autophagy to obtaining optimal fruit quality in each consuming stage.

## Supplementary Materials

The following are available online at https://www.mdpi.com/2073-4409/9/1/106/s1, Figure S1: Transversal semi-thin sections of pericarp pepper fruits. Green pepper var. Galena (A and B). Yellow pepper var. Galena (C and D). Red pepper var. Alonso (E and F). Asterisks indicate the areas studied under the TEM. Scale bars A, C, and E = 100 µm and B, D, and F = 50 μm. Table S1: Sequences of primers used in the RTqPCR analysis.
