# Addendum: López-Vidal et al. Is Autophagy Involved in Pepper Fruit Ripening? *Cells* 2020, *9*, 106

**DOI:** 10.3390/cells10071766

**Published:** 2021-07-13

**Authors:** Omar López-Vidal, Adela Olmedilla, Luisa María Sandalio, Francisca Sevilla, Ana Jiménez

**Affiliations:** 1 Department of Stress Biology and Plant Pathology, CEBAS-CSIC, 30100 Murcia, Spain; omalopez17@yahoo.com (O.L.-V.); fsevilla@cebas.csic.es (F.S.); 2 Department of Biochemistry, Cellular and Molecular Biology of Plants, EEZ-CSIC, 18160 Granada, Spain; adela.olmedilla@eez.csic.es (A.O.); luisamaria.sandalio@eez.csic.es (L.M.S.)

The authors would like to make an addendum to their published paper [1].

In the version of this article originally published, a key funding source was omitted, so the authors wish to add the funding project “RED2018-102397-T”. 

The change does not affect the scientific results. 

The rest of the manuscript does not need to be changed. 

The authors would like to apologize for any inconvenience caused.

## References

[1] López-Vidal O., Olmedilla A., Sandalio L.M., Sevilla F., Jiménez A. (2020). Is Autophagy Involved in Pepper Fruit Ripening?. Cells.

