# Supplementary material for: Is Autophagy Involved in Pepper Fruit Ripening?
Source: Cells. 2020 Jan 1;9(1):106. doi: 10.3390/cells9010106 (PMC7016703; doi:10.3390/cells9010106)
Supplement: Supplementary file 1 [file cells-09-00106-s001.zip › Table S1.pdf]

Table S1. Sequences of primers used in the RTqPCR analysis.

Name	Locus	Sequence 5´-3'	Primer efficiency (%)	Amplicon Dissociation T[a]
**Primers**				
S-CaATG4	LOC107874490	AGATGCTTGTTGCTCAGGCT	112.6	79.9°C
AS-CaATG4	LOC107874490	CTTTTGCTGCGTGCTAATGTT		
S-Ca-ATG5	LOC107857534	GGAGGAGTGGGAGGAAGTTC	109.1	79.2°C
AS-Ca-ATG5	LOC107857534	CCCAATTCGAGGCGCTAAAA		
S-CaATG9	LOC107870434	TCTTGTCCTTGACCCGCAG	110.2	77.7°C
AS-CaATG9	LOC107870434	GAAGTCCGCAATGAAACGC		
S-CaATG8a	AY486137.1	GAAGGTCCTGCTGTCCATCT	88.7	81.1°C
AS-CaATG8a	AY486137.1	AGACATGCCTGGAGACGATT		
S-CaLON1	LOC101264562	GATACCGGCTCATGTCATGC	88.0	74.8°C
AS-CaLON1	LOC101264562	GCAAGGCAGTCAACCAATCA		
S-CaLON2	LOC101256614	GCCAGCCCAGAACTTGAATT	84.9	77.3°C
AS-CaLON2	LOC101256614	CTCTCGCATCTGGTTTGAGC		
S-CaNBR1	LOC107875890	AGTGCGGTTGGTTTATCTGG	85.8	76.7°C
AS-CaNBR1	LOC107875890	TCGTTTTACCTGGGATCTGG		
S-CaACTIN	AY572427.1	TCTCCTGAAGAGCACCCTGT	85.2	77.4°C
AS-CaACTIN	AY572427.1	TACATGGCAGGGACATTGAA		
