# Supplementary figures and images for: Is Autophagy Involved in Pepper Fruit Ripening?
Source: Cells. 2020 Jan 1;9(1):106. doi: 10.3390/cells9010106 (PMC7016703; doi:10.3390/cells9010106)

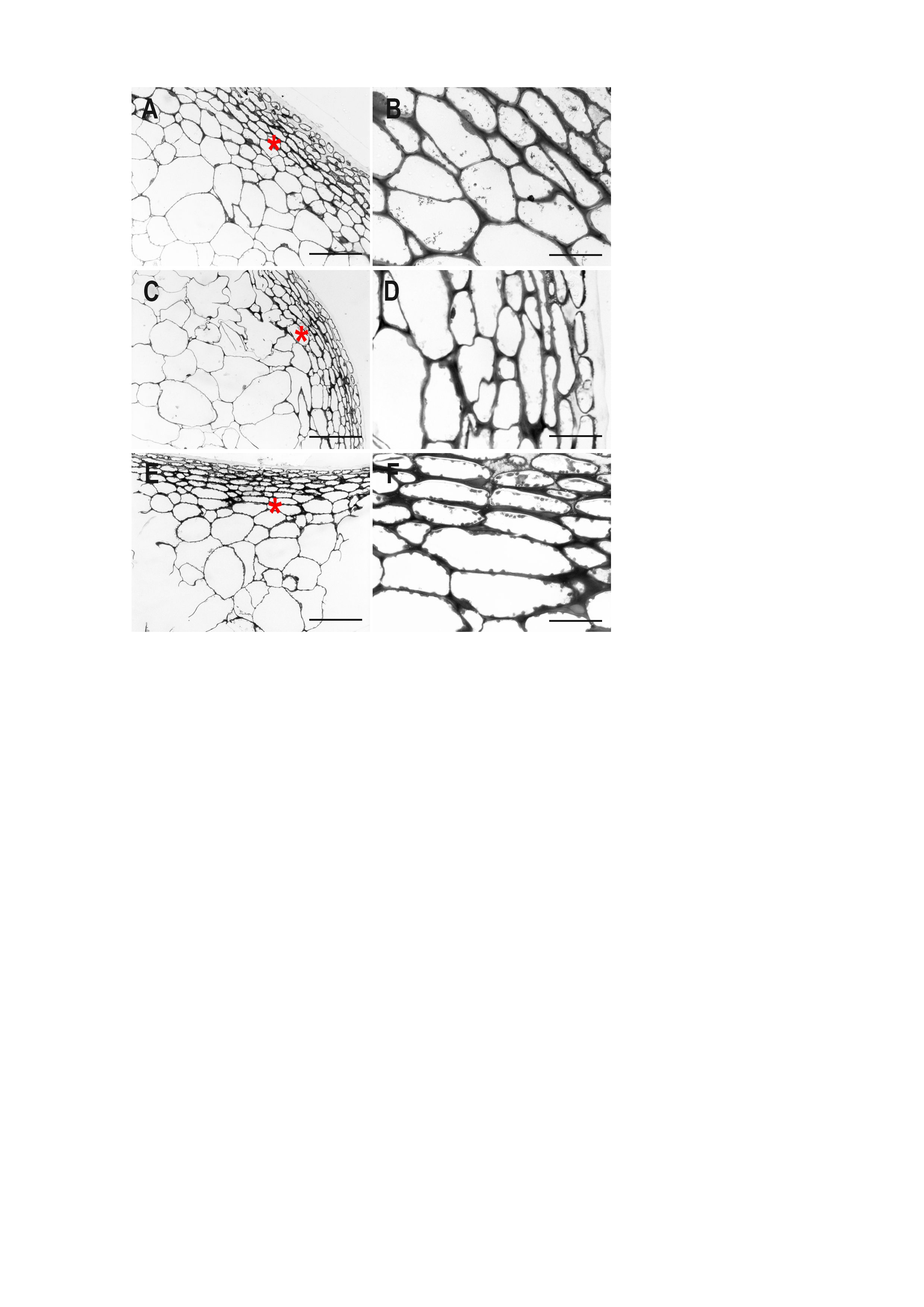

Supplement: Supplementary file 1 [file cells-09-00106-s001.zip › Fig S1.tif]
